# More than Simple Parasites: the Sociobiology of Bacteriophages and Their Bacterial Hosts

**DOI:** 10.1128/mBio.00041-20

**Published:** 2020-03-10

**Authors:** Patrick R. Secor, Ajai A. Dandekar

**Affiliations:** a Division of Biological Sciences, University of Montana, Missoula, Montana, USA; b Center for Translational Medicine, University of Montana, Missoula, Montana, USA; c Center for Biomolecular Structure and Dynamics, University of Montana, Missoula, Montana, USA; d Department of Microbiology, University of Washington, Seattle, Washington, USA; e Department of Medicine, University of Washington, Seattle, Washington, USA; University of Texas Health Science Center at Houston

**Keywords:** bacteria, bacteriophage, cheater, cooperation, sociobiology

## Abstract

Bacteria harbor viruses called bacteriophages that, like all viruses, co-opt the host cellular machinery to replicate. Although this relationship is at first glance parasitic, there are social interactions among and between bacteriophages and their bacterial hosts. These social interactions can take on many forms, including cooperation, altruism, and cheating. Such behaviors among individuals in groups of bacteria have been well described. However, the social nature of some interactions between phages or phages and bacteria is only now becoming clear. We are just beginning to understand how bacteriophages affect the sociobiology of bacteria, and we know even less about social interactions within bacteriophage populations. In this review, we discuss recent developments in our understanding of bacteriophage sociobiology, including how selective pressures influence the outcomes of social interactions between populations of bacteria and bacteriophages. We also explore how tripartite social interactions between bacteria, bacteriophages, and an animal host affect host-microbe interactions. Finally, we argue that understanding the sociobiology of bacteriophages will have implications for the therapeutic use of bacteriophages to treat bacterial infections.

## Abstract (precis)

Bacteria harbor viruses called bacteriophages that, like all viruses, co-opt the host cellular machinery to replicate. Although this relationship is at first glance parasitic, there are social interactions among and between bacteriophages and their bacterial hosts. These social interactions can take on many forms, including cooperation, altruism, and cheating. Such behaviors among individuals in groups of bacteria have been well described. However, the social nature of some interactions between phages or phages and bacteria is only now becoming clear.

## INTRODUCTION

Bacteria have traditionally and primarily been studied as single-celled organisms, with the implicit idea that populations of bacteria would behave as a collection of single-celled individuals. Over the last several decades, this paradigm has changed. It is now well recognized that bacteria can interact and engage in social behaviors such as cooperation (1, 2). Bacteria are also known to exist in complex, structured communities (3). These features of bacterial growth can intersect: growth in biofilms or cell aggregates can require the production of shared (or potentially shared) factors by individuals, and these factors also can confer nongenetic benefits on members of the group (4–6).

**FIG 1:**
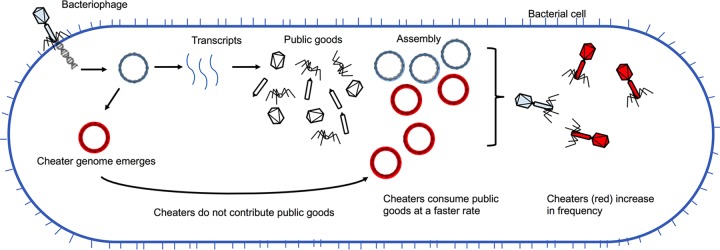
Pools of public goods provide incentive for bacteriophages to cheat. As bacteriophages replicate inside an infected cell, pools of public goods such as enzymes and capsid proteins are produced. Bacteriophages, like all viruses, have a high mutational rate, and cheaters can emerge that either do not contribute to public-good production or consume public goods (i.e., assemble complete virions) at a higher rate than ancestral bacteriophages. When the incentive to cheat is sufficiently high, cheater populations (shown in red) can expand.

Wherever there are bacteria, there are bound to be bacteriophages, bacterial viruses that constitute the most abundant collection of biological entities on the planet (7). Like other viruses, bacteriophages use host cellular machinery to replicate. Some bacteriophages are lytic and lyse their host after replicating while others can enter a lysogenic life cycle in which the bacteriophage genome integrates into the bacterial chromosome as a prophage, which is then replicated as the bacterial host divides. In response to certain signals or environmental factors, the prophage can be induced, initiating lytic replication. Other bacteriophages, such as filamentous *Inoviruses*, are capable of replicating without lysing their bacterial host; rather, virions are continuously extruded from the cell without causing lysis (8). Similar to the prior asocial dogma regarding bacteria is the conception of bacteriophage infection as a parasitism of individual cells. However, there is emerging evidence that bacteriophages modulate cooperative behaviors of their bacterial hosts. Furthermore, bacteriophages replicating within individual bacterial cells must cooperate and share a pool of capsid proteins, enzymes, and host products (Fig. 1). These pools of common goods provide opportunities for bacteriophages to exploit the cooperative behaviors of their kin.

## THE PROBLEM OF COOPERATION

One of the most well-studied types of group behavior in bacteria is cooperation (9). Cooperative behaviors imply a gain in benefit for the population at some cost to the individuals involved in the cooperative activity. Thus, the overall fitness of an individual, although potentially decreased by participation in the cooperative behavior, may be increased by activities of others in the group (10). One commonly studied type of cooperation involves the production of public goods, “a resource that is costly to produce and provides a benefit to all the individuals in the local group or population” (2). In the case of bacteria, these public goods are often shared exoproducts whose benefit accrues to the entire population, not to just the producing individual. Examples of bacterial public goods include exoproteases and iron-scavenging molecules called siderophores (11).

Cooperative behaviors among organisms of all taxa present a paradox for evolutionary ecology (12, 13) because incipient cooperation comes at a cost to the individual. Because noncooperators have a potential fitness advantage over cooperators, it is difficult to imagine how, in a well-mixed environment, cooperative behaviors could develop over evolutionary time. Cheaters in any community, if left unchecked, have the potential to cause the loss of cooperation from the population, and in some cases, this results in a tragedy of the commons (14).

## QUORUM SENSING, SIDEROPHORES, AND COOPERATION IN BACTERIA

Two of the best-described potentially cooperative behaviors of bacteria involve the activation of quorum sensing (QS) gene-regulatory circuits and the production of siderophores. Some bacteria use QS to regulate the production of public goods (reviewed in reference 15). QS circuits involve the production of an autoinducer signal by individual bacteria. This signal accumulates with increasing cell density and at a certain concentration binds to a cognate receptor, resulting in coordinated gene expression in the population (16). The general idea of using QS to regulate the production of public goods is that the production of these goods is synchronized among the population, a feature that may be particularly beneficial for shared products such as antimicrobials, where asynchronous production might lead to antimicrobial resistance in populations of competing microbes (17).

In one type of QS, Gram-negative bacteria produce acyl-homoserine lactone signals that bind to transcription factor receptors of the LuxR family (18). These LuxR homologs usually activate a set of genes, such as those encoding the bioluminescence operon *luxABCDE* in Vibrio fischeri, in which this type of QS was initially described (19). Light production is a type of public good, as are many QS-regulated products in other bacteria (20). There are other types of QS circuits: for example, cell-surface receptors in *Vibrio* recognize both acyl-homoserine lactone signals and a furanosyl borate diester, resulting in repression of a QS regulator (21). This regulatory scheme results in virulence gene expression at low cell densities in Vibrio cholerae. Another type of QS system, the *agr* system of staphylococci, involves a peptide signal called autoactivating peptide (AIP) (22). The AIP signal is transduced by the AgrCA two-component regulatory system, in which the response regulator AgrA activates transcription of a regulatory RNA called RNAIII, which ultimately derepresses transcription of several toxins.

In the opportunistic pathogen Pseudomonas aeruginosa, QS transcription factors regulate the production of a host of extracellular products, including antimicrobials, virulence factors, exopolysaccharides, and extracellular proteases (23), and the QS system of this bacterium has been widely studied as a cooperative system. When P. aeruginosa is grown on casein or bovine serum albumin as the sole carbon source, the production of a shared QS-regulated exoprotease, elastase, is required to break the protein down into constituent peptides and amino acids that individuals can use for carbon and energy. In well-mixed populations grown on casein or bovine serum albumin, there should be a strong incentive for individuals to mutate to avoid the metabolic burden of QS-regulated gene activation, and, in fact, QS mutants readily arise under these conditions (24, 25). Under some circumstances, QS mutants can reach a frequency in the population such that there is no longer a sufficient number of cooperators to produce enough public goods, a type of tragedy of the commons (26). However, P. aeruginosa and other bacteria have genetically encoded mechanisms that can deter social cheating (10, 27, 28). For example, the redox-active phenazine pyocyanin restricts social cheating in P. aeruginosa by selecting for individuals with intact QS systems, which are required to induce antioxidant defense systems that offset phenazine toxicity (29).

QS is not the only well-studied model of cooperation and conflict in bacteria. Another type of shared good are siderophores, iron-scavenging molecules produced by many species of bacteria. Siderophores called pyoverdines produced by P. aeruginosa bind to iron, and the iron-siderophore complex binds to a specific pyoverdine receptor. There are several pyoverdines and also several receptors among P. aeruginosa strains (30, 31). The diversity of pyoverdine molecules and of their receptors in pseudomonads generates several possible social interactions between competing strains of bacteria.

In settings where iron is limited, siderophore production presents a metabolic burden to individuals but a benefit to the population. When P. aeruginosa is grown in such settings, pyoverdine mutants emerge rapidly and can come to compose the majority of cells in the culture (9). These pyoverdine mutants, like the QS mutants discussed in the examples above, are cheaters that benefit from the pyoverdine produced by cooperators because expression of siderophore receptors is not dependent on expression of pyoverdine (32). By analogy, in mixed communities of *Pseudomonas* species, individuals expressing several siderophore receptor types can co-opt siderophores produced by others whether or not they are producing their own pyoverdine (31).

In the studies discussed above, a single public good under well-mixed conditions incentivizes cheating behaviors which can result in population decline or collapse. How then are cooperative behaviors maintained in microbial populations under natural settings? A recent study examined how different social selective pressures such as nutritional limitation and siderophore production might interact with each other to influence population outcomes (33). When P. aeruginosa is grown under a nutritional condition that requires QS for growth and under which iron is also limited, both siderophores and QS are required for maximal fitness of the population, but there are incentives for cheating from each behavior. Indeed, cheaters of both types readily emerged in this system. However, in this example a QS cheater is also a siderophore cooperator while siderophore cheaters participate in QS. The combined cooperative behaviors of the cheaters ultimately stabilize each other, preserving cooperative behaviors in the population.

## BACTERIOPHAGE SOCIOBIOLOGY

In the context of public-good cooperation, bacteriophages are not obvious contributors to this kind of social behavior. At a first approximation, bacteriophages are genetic parasites that usurp the genome and protein assembly infrastructure of the host cell to facilitate their own replication. However, the story is considerably more complex. In the context of their own replication, host cell-produced constituents of the bacteriophage virion can be thought of as public goods, giving bacteriophages an incentive to cheat (Fig. 1). In addition, bacteriophages have evolved mechanisms to exploit bacterial quorum sensing to make replication decisions or to expand or suppress bacterial cheater populations. Understanding the social interactions between bacteria and bacteriophages has implications for human health and disease, particularly for efforts to develop bacteriophages as therapeutics to treat multidrug-resistant bacterial infections (34, 35).

## BACTERIOPHAGES EXPLOIT PUBLIC GOODS

Perhaps one of the first observations of cheating by a bacteriophage was in early work characterizing the molecular biology of filamentous M13 bacteriophages that infect Escherichia coli. Like other *Inoviruses*, M13 virions are continuously extruded from their hosts, typically without causing lysis (8). In the 1970s, Griffith and Kornberg observed that truncated virions 20 to 50% the size of full-length M13 readily accumulate in serially passaged cultures (36). These miniphages package truncated genomes that are missing most or all open reading frames; only structural genetic elements, such as the origin of replication and packaging signal, remain, allowing M13 miniphage genomes to replicate and be packaged into M13 minivirions. M13 miniphages are obligate cheats (37); in the absence of full-length bacteriophages, M13 miniphages are unable to replicate. This fitness disadvantage stems from the inability of M13 miniphages to produce essential public goods such as capsid proteins or bacteriophage-encoded enzymes. In order to replicate, M13 miniphages must cheat and consume capsid proteins and other public goods produced by a minority population of full-length M13 bacteriophages coinfecting the same cell.

M13 miniphage cheaters, which harbor the minimum genetic elements required for replication, have proven to be of substantial benefit to human society. M13 miniphages gave rise to some of the first phagemids and other cloning vectors, tools that have been fundamental in our understanding of the basic principles of molecular biology (38, 39).

**FIG 2:**
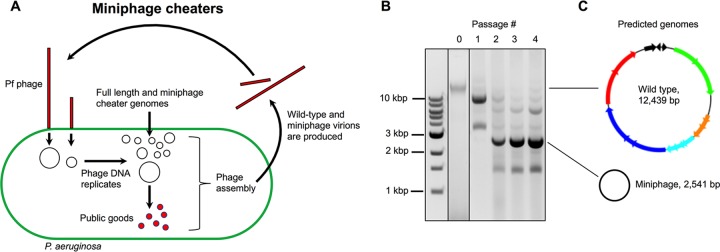
Pf4 miniphage cheaters emerge in serially passaged cultures. (A) Model depicting how Pf miniphages could emerge and propagate. (B) Episomal Pf DNA was isolated from P. aeruginosa cultures infected by serially passaged Pf. A dominant and stable subpopulation of miniphages with a 2.5-kbp genome was established after the second passage. (C) The dominant Pf miniphage genome is predicted to be composed of only intergenic features required for DNA replication and packaging into phage particles, similar to features of M13 miniphages. All other phage components such as capsid proteins are provided by low copy numbers of full-length phage coinfecting the same cell.

Many *Bacteria*, and even some *Archaea*, are infected by filamentous *Inoviruses* similar to M13 (40) and all of these bacteriophages are likely susceptible to cheating. For example, we observe that miniphages arise in serially passaged populations of Pf4, an *Inovirus* that infects P. aeruginosa PAO1 (Fig. 2). The dominant Pf4 miniphage genome we observed is ∼2.5 kbp. Because intergenic regions account for 2,541 bp of the full-length 12,439-bp Pf4 genome (41), these results suggest that Pf4 miniphages, like M13 miniphages, are missing most, if not all, open reading frames and that they must cheat in order to replicate.

Other bacteriophages, such as ϕ6, an RNA *Cystovirus* that infects Pseudomonas phaseolicola, cheat by consuming public goods such as capsid proteins at a higher rate than they contribute to their production. When propagated in the lab at a high multiplicity of infection (MOI; the number of virions per bacterial cell), ϕ6 cheaters emerge that display enhanced within-host fitness (42). This fitness advantage is realized only when the initial frequency of cheating bacteriophages is low. When cheater frequencies are high, ϕ6 cheaters have reduced fecundity, preventing them from sweeping through the bacteriophage population and causing a tragedy of the commons (42).

The molecular details of how ϕ6 cheats are not fully understood. One possible mechanism is that ϕ6 cheaters harbor a duplicated or an otherwise mutated *pac* region, which regulates genome encapsidation (42). Mutations in the *pac* region may enhance ϕ6 genome affinity for capsid proteins, allowing cheaters to replicate and consume public goods at a higher rate than ancestral wild-type bacteriophages. ϕ6 bacteriophages may also cheat by manipulating various stages of the lytic life cycle. For example, cheater bacteriophages may initiate cell lysis at too early a time point, before noncheating bacteriophages have matured. However, experimental evidence for this type of cheating is lacking (42, 43).

As mentioned above, at high densities, ϕ6 cheaters have reduced fecundity. This is in contrast to Pf and M13 miniphages, which rapidly achieve high population densities (Fig. 2). The fitness differences of these different species of cheater bacteriophages could be explained by density-dependent fitness benefits. Because ϕ6 cheaters are essentially the same size and composition as cooperator ϕ6 virions, pools of public goods would sustain only so many cheater virions, which would prevent high cheater population densities from being achieved. Conversely, Pf and M13 miniphages are only a fraction of the size of full-length cooperator bacteriophages, which may allow more cheater virions to be produced from a given pool of public goods, allowing miniphage cheaters to achieve higher population densities. Future investigations into density-dependent fitness benefits of various bacteriophage cheating strategies in relation to the tragedy of commons may reveal new ways to leverage bacteriophages as therapeutic tools to treat or prevent bacterial infections.

## ALTRUISM WITHIN BACTERIOPHAGE POPULATIONS

In the evolutionary arms race between bacteria and bacteriophages, bacteria have evolved diverse bacteriophage defense mechanisms (reviewed in reference 44). One example is CRISPR-Cas, a bacterial adaptive immune system that is present in approximately half of known bacterial genomes (45, 46). CRISPR-Cas systems are composed of arrays of short, ∼30-nucleotide repeats and spacers. The acquisition of spacers derived from unique genetic sequences present in infecting bacteriophages provides a memory of past infections, allowing swift immunity against future infections by bacteriophages harboring the same sequences.

Some bacteriophages attempt to evade this immune system by encoding anti-CRISPR (Acr) proteins (47). However, infection by a single bacteriophage is often not sufficient to suppress CRISPR-Cas immunity; Acr-dependent CRISPR-Cas suppression is often achieved only after multiple failed bacteriophage infections deliver a sufficient dose of Acr proteins to a recipient bacterial cell (48, 49). These failed bacteriophage infections can be construed to represent a form of altruism: bacteriophages that initiate failed infections suffer a cost in suppressing CRISPR-Cas immunity while kin bacteriophages stand to benefit by initiating successful infections. Recent work also demonstrates that Acr-encoding bacteriophages benefit Acr-negative bacteriophages by suppressing the evolution of CRISPR-mediated bacteriophage resistance, allowing Acr-negative phages to replicate in susceptible subpopulations of an otherwise resistant bacterial host (50).

It is interesting to speculate that a rapidly expanding bacteriophage population (such as the cheater bacteriophages discussed above) could serve as fodder to deliver Acr proteins to recipient bacterial cells. In addition to suppressing CRISPR-Cas immunity, bacteriophages initiating failed infections would be removed from the population, perhaps providing a mechanism to keep cheater (or any rapidly expanding) bacteriophage populations in check. It is also possible that infection by defective bacteriophages, which readily arise in culture but are unable to complete their life cycle (51), could display altruistic behaviors to subvert bacteriophage defense mechanisms. For example, defective bacteriophages could still deliver Acr genes to a host cell to overwhelm CRISPR defenses. Defective bacteriophages could also serve as decoys to subvert CRISPR-based immunity. However, defective bacteriophages can also work against bacteriophage populations by driving the acquisition of spacer sequences from defective phages, thus promoting CRISPR-based immunity (52). With the discovery of a diverse array of bacteriophage defense mechanisms (53, 54), understanding how bacteriophages subvert bacterial immune systems through the lens of sociomicrobiology may reveal new ways to expand or suppress bacteriophage populations, which may have implications for phage therapy and industrial applications.

## BACTERIOPHAGES, BACTERIA, AND TOXIC ALTRUISM

Some bacteriophages encode gene products that constitute bacterial public goods. For example, some pathogenic E. coli bacteria are lysogens for lambdoid Stx prophages that encode Shiga toxin. Shiga toxin provides bacterial populations with the means to defend themselves against phagocytic predators such as protozoans or neutrophils (55). Shiga toxin provides nonspecific protection to the bacterial population from predation by phagocytes and can be considered a public good. Because its production requires a lytic bacteriophage, Shiga toxin presents a kind of double-edged sword for cooperating bacterial populations. Shiga toxin is produced during lytic replication, and release of the toxin is accompanied by the death of the producer and simultaneous release of infectious Stx virions (56). A meta-analysis of the Stx literature revealed that under more natural conditions (i.e., not conditions such as high concentrations of mitomycin C), Stx prophage induction occurred in approximately 1% of the bacterial population (57). However, the low level of Stx induction was accompanied by the release of relatively large amounts of toxin (58, 59).

Under the threat of predation by phagocytes, bacteria not infected by Stx could be considered cheaters as they do not produce Shiga toxin. These cheaters, which can include commensal strains of E. coli (60), are susceptible to infection by Stx bacteriophages and can be forced to cooperate. After infecting a cheater, Stx can enter a lytic or lysogenic life cycle. If Stx enters the lytic cycle, cheaters are converted into Shiga toxin producers and are subsequently lysed and removed from the population. Alternatively, if cheaters are lysogenized by Stx, Shiga toxin genes are preserved in the bacterial population, contributing to the maintenance of this cooperative behavior (56).

## BACTERIOPHAGES MODULATE BACTERIAL CHEATER POPULATIONS

**FIG 3:**
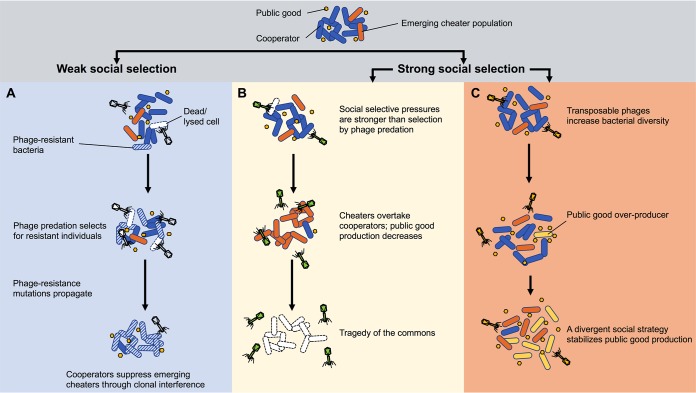
Bacteriophages can suppress, expand, or stabilize bacterial cheater populations. Strong social selection here refers to a selective pressure (such as iron limitation) that promotes the expansion of bacterial cheater populations. (A) When selection for bacteriophage-resistant individuals is stronger than selection for cheaters (red cells), bacteriophage resistance mutations (stripped cells) propagate at a higher rate through the numerically dominant cooperator population (blue cells) than in the emerging cheater population. (B) When the incentive to cheat outweighs the selective pressures of bacteriophage predation, cheater populations can expand to the point of population collapse. (C) Transposable bacteriophages randomly integrate into the bacterial chromosome and drive bacterial diversification. Coupled with a strong social selective pressure, infection by a transposable bacteriophage can promote a divergent social strategy wherein both cheaters and public-good overproducers emerge to stabilize the bacterial population.

Strong social selective pressures allow bacterial cheater populations to expand. Depending on whether social selective pressures are strong or weak, bacteriophages can expand or suppress bacterial cheater populations. For example, predation by the lytic phage SBW25Φ2 imparts a continual selective pressure on Pseudomonas fluorescens that promotes the emergence of bacteriophage-resistant individuals (61). When cooperation is the dominant strategy within the population (i.e., the cooperator population is numerically dominant), bacteriophage-resistant individuals are far more likely to emerge in cooperator populations than in cheater populations, and bacteriophage resistance genes propagate through the cooperator population. If the fitness benefits conferred by bacteriophage resistance mutations outweigh the benefits of social selective pressures (e.g., iron limitation), then bacteriophage-resistant cooperators suppress cheaters by clonal interference, i.e., the competition of two beneficial mutations (phage resistance mutations and siderophore inactivating mutations) in the same genetic background (Fig. 3A) (61). Of course, if cheaters were numerically dominant, predation by these same bacteriophages would be expected to suppress a minority cooperator population through the same mechanism, perhaps resulting in population decline or crash due to a lack of public-good production. However, it is worth pointing out that high densities of cheater bacteriophages could be accompanied by reduced fecundity/fitness, which would have dramatic impacts on the modulation of bacteria cheater populations by bacteriophages.

Lysogenic bacteriophages can also select for populations of cooperators. In P. aeruginosa, QS-null individuals (Δ*lasR* Δ*rhlR*) were more susceptible to infection by lysogenic bacteriophages (strains JBD30 and D3112) than wild-type bacteria with intact QS systems (62). It was observed that lysogenic bacteriophages preferentially adsorb to and infect QS-null individuals. While the mechanistic details remain to be worked out, QS inactivation could promote bacteriophage attachment to QS-null individuals by inducing the expression of cell surface molecules important for bacteriophage attachment (type IV pili in this case) or by modulating the production of QS-regulated capsule components that interfere with bacteriophage attachment. Because type IV pilus-mediated twitching motility is similar between wild-type and QS-null P. aeruginosa bacteria (63), differences in bacteriophage adsorption to wild-type and QS-null bacteria are likely not explained by differential expression of a cell surface receptor. Surface hydrophobicity measurements revealed a higher hydrophobicity of wild-type P. aeruginosa than QS-null P. aeruginosa, raising the possibility that differential capsule compositions between wild-type and QS-null strains affect susceptibility to bacteriophage infection.

In the above example, bacteriophages selected for cooperative behaviors in bacterial populations. However, under iron-limiting conditions (a strong social selective pressure), bacteriophage predation can promote the expansion of bacterial cheater populations. This is apparent in iron-limited P. aeruginosa cultures infected by lytic LKD16 bacteriophages. Under such conditions, bacteriophage predation was insufficient to suppress bacterial cheater populations, and cheater populations invaded to the point of population decline due to lack of sufficient siderophore production (Fig. 3B) (64).

Transposable bacteriophages add yet another layer of complexity to bacteriophage-bacterium social interactions. In an epidemic strain (LES) of P. aeruginosa, the *mu*-like transposable bacteriophage LESϕ4 randomly integrates into the chromosome, driving bacterial adaptation and diversification (65). Under iron-limiting conditions, LESϕ4 had higher insertional frequencies in genes related to siderophore production and PQS quorum sensing than in iron-replete cultures (66). The inactivation of these social genes allowed a cheater population to expand under iron-limiting conditions. Interestingly, LESϕ4 infection did not cause a tragedy of the commons or population decline under iron-limiting conditions. Rather, a divergent social strategy emerged: in addition to cheaters not producing siderophores, a siderophore-overproducing population also emerged, stabilizing siderophore production (Fig. 3C).

## BACTERIOPHAGES SENSE AND RESPOND TO SOCIAL CUES

Transduction of genetic material by bacteriophages is a major mechanism of horizontal gene transfer within and between microbes (67), and some bacteriophages have acquired genes that facilitate social interactions with bacteria. For example, bacteriophages that infect *Clostridium* species encode homologs of the *agr* quorum system (68) and QS response regulators (69). Some *Vibrio* bacteriophages encode functional QS receptor genes that are homologous to their bacterial counterparts that sense and respond to bacterial autoinducers (70, 71). In addition, bacterial QS autoinducers can induce lytic bacteriophage replication in bacteria isolated from soil and groundwater (72). How QS genes carried by bacteriophages integrate into larger social networks in bacterial populations is only beginning to be understood.

The QS receptors encoded by *Vibrio* bacteriophages are among the most well-understood. *Vibrio* bacteriophage QS receptors are functional and respond to bacterial autoinducers to regulate lysis/lysogeny decisions (70, 71). When bacteriophages sense autoinducers produced by a quorum of bacteria, lytic replication is initiated to capitalize on a bacterial population full of potential hosts.

Other bacteriophage-encoded QS genes include autoinducer synthetases (68). It is possible that bacteriophages encoding these genes produce bacterial autoinducers to augment or prematurely activate bacterial QS signaling. This could be a mechanism whereby bacteriophages force their hosts to cooperate and produce or even overproduce public goods, allowing nonproducing cheaters to emerge, similar to the divergent social strategy discussed above in P. aeruginosa infected with transposable bacteriophages under iron-limited conditions (66).

In some cases, rather than listening in or contributing to microbial conversations, bacteriophages may opt to kill the conversation. The *Iodobacter* bacteriophage ϕPLPE may do this as it encodes a predicted acylhydrolase (73), which could block QS signaling by degrading acylhomoserine lactone molecules. Bacteriophage-encoded enzymes that degrade QS autoinducers could delay the activation of QS-regulated genes in some circumstances, producing a temporary cheater population.

Last, many bacteriophages encode their own fully functional and independent QS-like system, called arbitrium (74, 75). Like QS, arbitrium signaling uses unique peptide autoinducers and cognate receptors. One purpose of the arbitrium system is to sense how many bacteria in a given population are infected by kin bacteriophages in order to guide lysis/lysogeny decisions. As with bacterial QS, these decisions are mediated by peptide autoinducer concentrations; as the peptide accumulates, lytic replication is inhibited, and the lysogenic life cycle is promoted. Like QS, arbitrium signaling is probably susceptible to exploitation by both bacteriophages and bacteria. For example, other species of bacteriophages may encode arbitrium orphan receptors that allow them respond to arbitrium signals produced by competing bacteriophages. Conversely, arbitrium receptor genes may become inactivated in some bacteriophages, producing a cheater subpopulation. However, arbitrium cheaters that ignore the peptide signal may enter but never exit the lytic life cycle could thus cause the collapse of the host bacterial population (which would be beneficial in the setting of infectious disease). While arbitrium-signaling peptides have therapeutic potential to induce endogenous prophages to kill infecting bacteria, endogenous prophage induction could have unintended consequences, as we discuss in the following section.

## TRIPARTITE INTERACTIONS BETWEEN BACTERIOPHAGE, BACTERIA, AND ANIMALS

Bacteriophage virions play diverse and surprising roles in tripartite interactions between bacteriophage, bacteria, and animals. In some cases, bacteriophage virions may act as a public good themselves. Filamentous *Inoviruses* are a prime example; these relatively stiff, negatively charged rods can bind to and sequester cationic antimicrobials such as antimicrobial peptides (76) and aminoglycoside antibiotics (77). The sequestration of antimicrobials by these bacteriophages allows bacteria to tolerate otherwise lethal doses of antimicrobials (77), which is associated with the emergence of strains of P. aeruginosa with fixed antimicrobial resistance in human infections (78). Thus, in cases in which *Inoviruses* provide bacterial populations protection against antimicrobials, the virions are a public good.

Pf bacteriophages also have immunomodulatory properties that could serve a social role in tripartite interactions. Pf virions directly stimulate a type I interferon antiviral response in human and mouse immune cells through the pattern recognition receptor Toll-like receptor 3 (TLR3) (79). TLR3 recognizes double-stranded RNA within endosomes (a membrane-bound vesicle). However, Pf bacteriophages package a single-stranded, circular DNA genome. While the precise mechanism for how Pf bacteriophages induce TLR3 signaling is unknown, preliminary observations suggest that Pf virions escape endosomes and that Pf DNA is transcribed by the eukaryotic cell (79). It is possible that Pf RNA forms hairpins that are then recognized by TLR3, but it is unclear how the Pf RNA would make it back into an endosome to be recognized by TLR3. An alternative possibility is that Pf virions, which are filaments with diameters (6 to 7 nm) and charge densities comparable to those of double-stranded nucleic acids (76), directly interact with TLR3 to induce antiviral responses. However, direct experimental evidence of such an interaction is lacking.

Stimulation of TLR3 signals through the adaptor protein TRIF, which then induces NF-κB-dependent transcription of genes involved in a type I interferon antiviral response (79). The antiviral response invoked by Pf virions suppresses bacterial clearance and is maladaptive in the face of a bacterial infection, benefiting P. aeruginosa as it establishes an infection. Thus, for P. aeruginosa, Pf virions may themselves be considered a public good. Intriguingly, it is possible that Pf miniphages could induce the same response, i.e., that “cheaters” in one context (phage replication) might still be a functional public good in another (P. aeruginosa infection). It will be interesting to see if Pf miniphages or other types of cheater bacteriophages are present at sites of infection.

Other bacteriophages also have immunomodulatory properties that could serve a tripartite social role. For example, in inflammatory bowel diseases, *Caudovirales* bacteriophage populations are expanded (80) and can directly stimulate a TLR9-dependent immune response that promotes inflammation (81). TLR9 recognizes CpG-containing microbial DNA (82). When *Caudovirales* bacteriophages (which package linear double-stranded DNA [dsDNA]) are internalized by immune cells, the bacteriophage DNA is released from the virion where it directly stimulates inflammation through TLR9 (81). Increased inflammation can contribute to dysbiosis of the gut microbiota and favor infection by pathogenic bacteria (reviewed in reference 83). Therefore, the immunomodulatory properties of *Caudovirales* bacteriophages could serve as a public good for enteric pathogens.

Bacteriophage-mediated immunomodulation has also been reported in bacterial symbionts. A recent study demonstrated that marine sponges, despite continuously filtering seawater containing abundant and diverse microbes, harbor a stable viral community that includes bacteriophages (84). While many of the bacteriophages that infect sponge symbionts are virulent (as evidenced by the enrichment of bacteriophage-defense mechanisms in sponge symbiont genomes [85]), some of these bacteriophages encode immunomodulatory proteins that alter host-microbe interactions. These immunomodulatory bacteriophage-encoded proteins contain ankyrin repeats, a widely distributed protein motif that facilitates diverse protein-protein interactions in prokaryotes and eukaryotes (86). Jahn et al. demonstrated that secreted bacteriophage-encoded ankyrin proteins suppressed inflammatory cytokine production and phagocytic bacterial uptake by the sponge immune system (84). The suppression of the sponge’s immune system by these so-called ankyphages facilitated symbiont-host coexistence. It would be interesting to know if bacteriophage-encoded ankyrins protected only the producing (bacteriophage-infected) bacteria from immune clearance or if bacteriophage-encoded ankyrin proteins serve a more social role that extended to the entire symbiont population.

Sponges are not the only example of ankyphages facilitating the tolerance and maintenance of bacterial symbionts within their hosts. While bacteriophage-encoded ankyrin proteins are prevalent among cellular organisms, they are relatively scarce among bacteriophages (84), with the notable exception of prophage WO in *Wolbachia* (87), the most widespread endosymbiotic bacteria on the planet (88). *Wolbachia* bacteria are maternally inherited obligate intracellular bacteria that infect arthropods and are perhaps most famous for their ability to manipulate the reproductive biology of their host. These manipulations include feminization, whereby males are converted into females, and cytoplasmic incompatibility (CI), whereby sperm from *Wolbachia*-infected males are unable to fertilize eggs from noninfected females. Ankyrin genes harbored by prophage WO (*pk1* and *pk2*) are correlated with feminization and CI in different arthropod hosts (89, 90). In addition to ankyrin proteins, prophage WO also expresses the non-ankyrin genes *cifA* and *cifB*, which mediate CI in Drosophila melanogaster (91, 92). These observations demonstrate that bacteriophage WO has evolved multiple ways to manipulate the reproductive biology of its arthropod hosts, which could help explain why *Wolbachia* bacteria (and bacteriophage WO) are so widespread in nature. Understanding tripartite social interactions between bacteriophage WO, *Wolbachia*, and arthropods could provide important insights into the development of *Wolbachia*-based strategies to control the transmission of vector-borne diseases such as dengue, Zika, and yellow fever virus infections.

## CONCLUDING REMARKS AND FUTURE LINES OF INVESTIGATION

Social cooperation and conflict are common in microbial populations. Like their bacterial hosts, bacteriophages exhibit complex social interactions and integrate into larger microbial social networks in ways that we are only beginning to understand. These social interactions impact the fitness and population outcomes for bacteriophages, bacteria, and, as is becoming clearer, animals.

Most sociobiological studies of bacteriophage-bacterium interactions have focused on cooperation and cheating. Bacteriophage manipulation of bacterial cheaters or communication systems like QS could modulate virulence factors, siderophores, proteases, and other such public goods, affecting the virulence capacity of the bacterial population, as previously suggested (62). These interactions, particularly in concert with the ability of some bacteriophages to subvert animal immune systems (79, 81, 84, 91), could have dramatic implications for the therapeutic use of bacteriophages to treat bacterial infections. For example, in some scenarios, it is possible to imagine that the therapeutic use of bacteriophages to treat bacterial infections could lead to bacterial population collapse by favoring the emergence of less virulent bacterial cheaters. Such bacteriophages could be termed “social killers” and would not necessarily need to be ultravirulent and produce large plaques on lawns of bacteria in a Petri dish. Thus, studies exploring the sociobiology of bacteriophages have the potential to expand the number and types of bacteriophages that could be used for phage therapy. On the other hand, it is also possible that bacteriophages could stabilize cooperative behaviors in the infecting bacterial population and modulate/suppress host immunity, thereby promoting infection. As phage therapy becomes more mainstream, it will be important to keep these social considerations in mind while hunting for therapeutic bacteriophages and while monitoring treatment efficacy.

Our review also emphasizes the idea that bacteria or bacteriophages as cheaters or cooperators is context specific and may not describe the totality of the interaction between individuals. Bacterial or bacteriophage cheaters may not really be cheaters *per se*. Rather, cheaters could instead be relieved from the burden of a costly communal task in order to explore alternative biological functions. For example, in settings where bacteriophages sequester antimicrobials, the antibiotic-resistant bacteria that emerge may have once been cheaters not producing bacteriophages.

The complexity of bacteriophage-bacterium social interactions also should suggest caution about the interpretation of data in experimental systems in which there is thought to be selective pressure on a cooperative trait, particularly in the design of synthetic biological systems that use bacterial QS to regulate the production of an exoproduct. In fact, it may be that there are overlaid pressures from bacteriophage-bacterium interactions that have the effect of altering the fitness benefit to the cheating individual. In the end, understanding how social interactions combine to yield a whole-population outcome will be critically important for a myriad of experimental and applied interactions between bacteriophages and their bacterial hosts.
